# Engineering principles for rationally design therapeutic strategies against hepatocellular carcinoma

**DOI:** 10.3389/fmolb.2024.1404319

**Published:** 2024-06-13

**Authors:** Alexis Hernández-Magaña, Antonio Bensussen, Juan Carlos Martínez-García, Elena R. Álvarez-Buylla

**Affiliations:** ^1^ Instituto de Ecología, Universidad Nacional Autónoma de México, Ciudad de México, Mexico; ^2^ Departamento de Control Automático, Cinvestav-IPN, Ciudad de México, Mexico; ^3^ Centro de Ciencias de la Complejidad (C3), Universidad Nacional Autónoma de México, Ciudad de México, Mexico

**Keywords:** hepatocellular carcinoma, cancer, rational design, cell plasticity, medical systems biology

## Abstract

The search for new therapeutic strategies against cancer has favored the emergence of rationally designed treatments. These treatments have focused on attacking cell plasticity mechanisms to block the transformation of epithelial cells into cancerous cells. The aim of these approaches was to control particularly lethal cancers such as hepatocellular carcinoma. However, they have not been able to control the progression of cancer for unknown reasons. Facing this scenario, emerging areas such as systems biology propose using engineering principles to design and optimize cancer treatments. Beyond the possibilities that this approach might offer, it is necessary to know whether its implementation at a clinical level is viable or not. Therefore, in this paper, we will review the engineering principles that could be applied to rationally design strategies against hepatocellular carcinoma, and discuss whether the necessary elements exist to implement them. In particular, we will emphasize whether these engineering principles could be applied to fight hepatocellular carcinoma.

## Introduction

**FIGURE 1:**
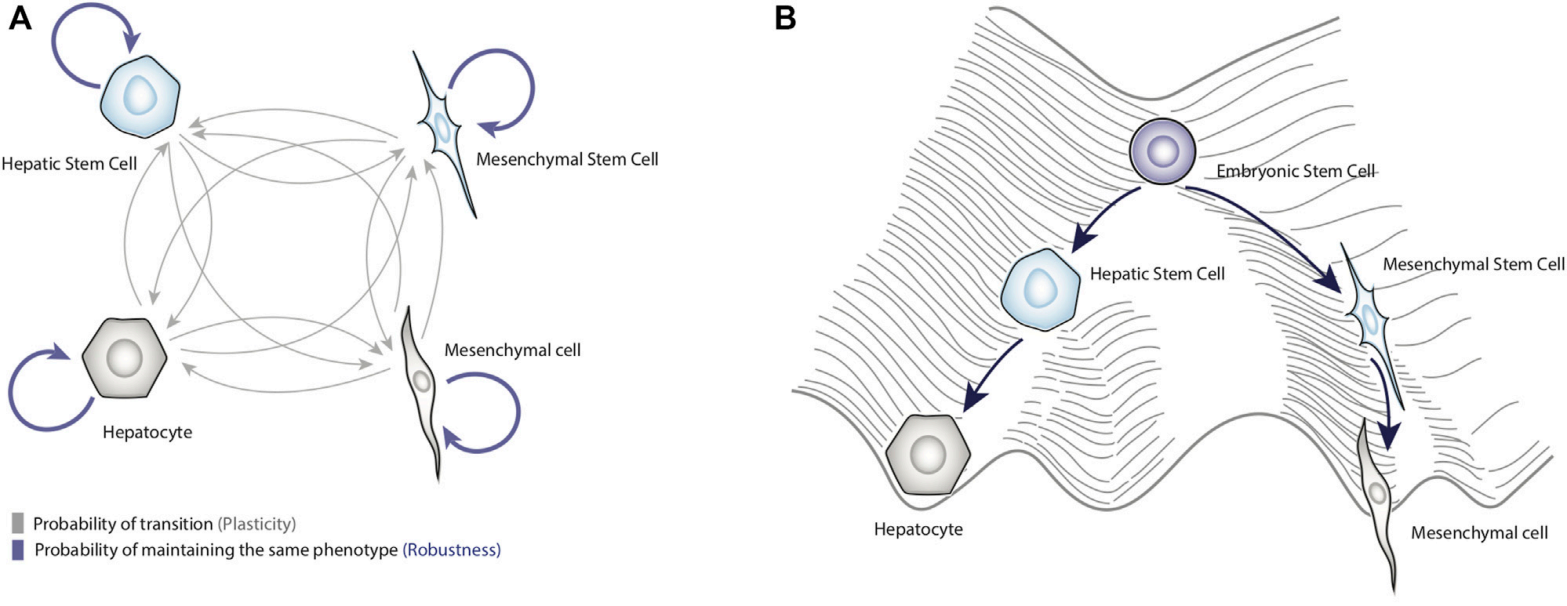
Dynamical properties of biological systems. Panel **(A)** shows a schematic representation of cell plasticity and biological robustness. The blue arrows represent the probability of remaining in a specific phenotype, and gray arrows represent the probability of transitioning to other phenotypes. Panel **(B)** represents Waddington’s conceptual model of the epigenetic landscape applied to liver cells. This figure shows how terminal phenotypes such as hepatocytes and mesenchymal cells can be obtained from a totipotent cell.

Currently, cancer is one of the most important chronic-degenerative diseases, since it is responsible for 10 million deaths per year (Ferlay et al., 2021). However, not all cancers affect patients in the same way; it has been reported that some neoplasms such as hepatocellular carcinoma (HCC) are particularly lethal (Rumgay et al., 2022). Thus, it is a priority to identify the molecular mechanisms involved in the malignant transformation of somatic cells and determine the factors that increase the mortality rate of cancers like HCC. In this sense, experimental reports have shown that epithelial-to-mesenchymal transition (EMT) is crucial to regulate the development of HCC (Yuan et al., 2019). Broadly speaking, EMT is a reversible process by which epithelial cells lose cell-cell adhesion, change their morphology, and acquire properties of mesenchymal cells such as migratory ability (Figure 1A) (Cicchini et al., 2015; Wickramaratne et al., 2021).

Recently, a novel therapeutic approach that rationally targets EMT plasticity has been proposed (Yang et al., 2019; Huang et al., 2022). This approach uses drugs to either redirect or block the EMT in patients (Huang et al., 2022). Unfortunately, this strategy has not achieved notable improvements in cancer therapy (Shi et al., 2023). It is thought that the underperformance of this strategy is due to the lack of a comprehensive understanding of mechanisms that regulate tumor cell plasticity (Yang et al., 2019; Boumahdi and de Sauvage, 2020; Wickramaratne et al., 2021). In consequence, it is essential to explore better design techniques capable of dealing with complex problems such as the regulation of EMT plasticity. In this sense, a discipline that has historically specialized in solving complex problems is engineering.

In fact, abstract engineering principles have been successfully applied to create new metabolic pathways and artificial biological processes with broad spectrum applications (Choi et al., 2019). Furthermore, the methodology of engineering has been used to optimize already existing metabolic, genetic, and signaling pathways (He et al., 2016). Interestingly, recent research has focused on rationally designing cancer treatments (Wu et al., 2019; Shih and Chen, 2022). In the present work, we will examine the challenges that a rational design against HCC should be able to face and we will analyze the clinical viability of this approach in our current context.

### Controlling biological systems according to engineering

The rational approach that engineering follows to solve problems begins with the creation of models that represent the system that is sought to be controlled (Ogata and Brewer, 1971; Mellodge, 2015). Then, control laws with inputs are proposed to regulate the system and guide it to a state or states that are of interest. Next, the parameters and materials necessary to implement the created control laws are analyzed. Subsequently, devices are built that implement the control laws with the proposed specifications, and finally, the performance of the solution is experimentally tested (Ogata and Brewer, 1971; Mellodge, 2015). This approach has passed integrally to metabolic engineering and synthetic biology, allowing the creation of highly complex *de novo* biological processes (Siuti et al., 2014; He et al., 2016). In these engineering applications, it is particularly important to know the dynamics of the flows of matter in the system, this in order to optimize already existing processes (Haellman et al., 2021).

To practically illustrate the principles mentioned above, Ajo-Franklin and colleagues noticed that some protein production systems in yeast gradually lost efficiency at the time the producing cells entered cell division (Ajo-Franklin et al., 2007). In consequence, this research team set out to design a protein production system with memory, that is, with the ability to maintain protein production regardless of cell division state. To do this, they conceptualized two genes, a controller gene and a process gene. The controller gene can only be induced with galactose as input, and once induced, it will express an artificial transcription factor fused to RFP, which activates the expression of a second gene. As a result of the above, the second gene expressed another artificial transcriptional factor fused to YFP, which is capable of inducing its own expression. The idea was that, when yeast transfected with this genetic system were induced with galactose, they would begin to produce YFP stably over time, regardless of whether they divided or not (Ajo-Franklin et al., 2007).

To ensure this behavior, the team represented the interactions of the system with a mathematical model and upon analyzing it, they found that the affinity constants of the transcriptional factors require certain specific values ​​to achieve the desired behavior. After several tests in the laboratory, they created the transcriptional factors with the desired parametric values, and thus they were able to construct their production system with the properties of automatically producing a protein of interest over time, without the need to become induced (Ajo-Franklin et al., 2007). The above demonstrates that, through the use of mathematical models, it is possible to control a biological system at the molecular level so that it performs a desired behavior.

### Therapeutic strategies to control hepatocellular carcinoma

In this direction, pioneering works have begun to implement engineering principles to create therapeutic strategies to eliminate HCC. From a control perspective, these treatments focus on destroying tumor cells and preventing metastasis (Wu et al., 2019). In particular, Han *et al* designed a series of genetic circuits with the ability to detect hallmark markers of HCC such as AFP activation, and interactions between YAP1 and 14-3-3s. The basic assumption was that these markers are notably increased in HCC cells compared to healthy cells, and by having high levels of these three input signals, their genetic circuits could express an antibody against VEGF in order to prevent tumor metastasis. This design was tested experimentally in mice, and it was found that there was a significant decrease in the size of HCC tumors in the diseased mice (Han et al., 2020).

Continuing with this line, Yang and Ding improved this idea and designed a genetic circuit capable of selectively eliminating HCC tumor cells (Yang and Ding, 2021). Furthermore, they added an extra layer of safety, coupling the expression of the anti-apoptotic Bcl-2 gene with the hBAX gene. The underlying motivation was to prevent premature activation of hBAX expression and accidently activate the apoptosis pathway, in case basal levels of YAP1 exist. The decisive test for this circuit was performed by mixing the IMR90 and PLC/PRF/5 cell lines, the first being from lung, while the second comes from liver carcinoma. In this experiment it was found that the circuit selectively killed PLC/PRF/5 cells, leaving lung cells undamaged (Yang and Ding, 2021). However, despite the promising results obtained *in vitro*, the circuit was not able to eliminate the entire population of cancer cells, which shows that it is necessary to improve the design before implementing *in vivo* applications (Yang and Ding, 2021). This same problem was found by Lin and colleagues, who designed a genetic circuit that used miR-196a and miR-126 as inputs to induce hBAX expression in mice with HCC (Lin et al., 2018). As proof-of-principle, these approaches demonstrate that it is possible to control HCC tumor cells. However, the way in which these control designs are conceptualized still needs to be improved, and to do so it could be necessary to delve deeper into the functioning of tumor cells.

### Understanding the state space of a biological system

In the example of Ajo-Franklin and colleagues, it can be seen that they guided their genetic system toward a specific state, and the task was relatively simple because the system was small (Ajo-Franklin et al., 2007). However, controlling complex systems such as the regulatory networks that underlie the cell plasticity (Huang et al., 2022; Shi et al., 2023) is complicated due to the multistability of these biological systems, that is, the existence of several operating stable states (i.e., attractors) (Laurent and Kellershohn, 1999). In this regard, one way to represent the biological multistability was introduced by Conrad Hal Waddington, who proposed a conceptual model, known as the “epigenetic landscape”, that represents all the possible differentiation routes that a totipotent cell could take (Rajagopal and Stanger, 2016a) (Figure 1B).

In this model, a stem cell represented by a ball, placed at the top of the landscape would roll down and reach the bottom of valleys that are equivalent to stable cell phenotypes (e.g., cell types or cell fates), (Ladewig et al., 2013; Jia et al., 2017) (Figure 1B); these routes through the epigenetic landscape corresponds to cellular differentiation pathways. Although the epigenetic landscape metaphor was originally proposed to illustrate development, it is also useful to depict phenotypic transitions (i.e., cell plasticity) occurring in adult tissues during regeneration or cancer progression (Rajagopal and Stanger, 2016b; Pisco and Huang, 2015). It has been shown that differentiated cells can also move towards another differentiated state, a process called trans-differentiation, for instance, epithelial-mesenchymal transition (Jopling et al., 2011). Also, differentiated cells can transit towards less differentiated states (i.e., dedifferentiation) (Jopling et al., 2011), for example, hepatocytes can convert into liver progenitor cells in response to chronic injury (Li et al., 2020); even differentiated cells can transit to a pluripotent state (i.e., reprogramming) in response to overexpression of transcription factors such as OCT4, SOX2, KLF4, and MYC (Jopling et al., 2011). In this context, the heights of hills represent the difficulty of jumping from one attractor to another (Pisco and Huang, 2015). As discussed later, the enormous regenerative capacity of the liver depends significantly on trans-differentiation and dedifferentiation processes.

Notably, these phenotypic transitions are constrained by an underlying gene regulatory network (GRN). For example, various regulatory circuits controlling the EMT have been described experimentally (Saunders and McClay, 2014; Migault et al., 2022). In broad terms, epithelial markers (such as mir34a, mir200b, miR00c) and mesenchymal markers (such as SNAI1/2, TWIST1/2, ZEB1/2, FOXC2) form two distinct modules or “teams” (Hari et al., 2022). It has been observed that nodes within the same “team” activate each other, while inhibition is observed among members of different “teams”, enabling a switch behavior during EMT (Hari et al., 2022) and promoting the stability of phenotypes, either epithelial or mesenchymal, once reached.

Although GRNs underlying biological processes are extremely complex, it is possible to characterize their dynamical behavior and quantify their associated epigenetic landscape with the help of mathematical models. In this sense, mathematical abstractions, like differential equations, can be used to represent experimental information about the regulation of nodes (e.g., genes, proteins, miRNAs) and explore quantitively Waddington’s epigenetic landscape (Karlebach and Shamir, 2008; Álvarez-Buylla Roces et al., 2018). The specific methodology for deriving the epigenetic landscape from dynamical GRN models has been reviewed previously (Davila-velderrain et al., 2015; Álvarez-Buylla Roces et al., 2018). Thus, from a dynamical systems perspective, the epigenetic landscape graphically represents the topography of the state space (i.e., the set of possible states of the system) of a GRN, where stable states are at the bottom of valleys and unstable states at the top of hills (Huang, 2013). According to the notion of stability, a cell will tend to maintain its phenotype despite the presence of relatively slight environmental fluctuations or noise in gene expression (Huang, 2013).

Importantly, theoretical-experimental evidence has proven that Waddington´s epigenetic landscape can be quantified at the genome scale. Specifically, Shi and collaborators analyzed several independent sets of RNA-seq data; for integrating the transcriptomic profiles with a protein-protein interaction network created from interactions reported in databases and using specialized algorithms to quantify the levels of network entropy, it was observed the appearance of stable gene expression patterns that corresponded to the terminal phenotypes of somatic cells (Shi et al., 2018). In addition, they were able to trace all the differentiation routes that pluripotent cells follow to reach terminal phenotypes such as hepatocytes (Shi et al., 2018).

These results open the possibility of using the epigenetic landscape as a blueprint to control cell plasticity. It would be used to explore alterations in gene expression favoring that a specific cell fate can be attained, for example, senescence, apoptosis, or any other state that inhibits proliferation, which could be a powerful tool against diseases such as cancer, particularly in HCC.

### Epigenetic landscape as a strategic blueprint

The next question to address is how to use information about the dynamics of biological systems as a design blueprint. The answer to this question may lie in recent advances in metabolic engineering. Specifically, Buldum and colleagues sought to improve the prediction of the yields of bioprocesses carried out by microorganisms (Buldum et al., 2020). To do this, they used as a case study the creation and optimization of a genetic circuit to induce the bacterial cellulose synthesis pathway in *Escherichia coli*. They used a GRN to integrate into the design all aspects of the biological multistability and molecular complexity of the pathway under natural conditions. With this approach, they were able to accurately predict and significantly improve the performance of transgenic bacteria (Buldum et al., 2020). Learning from the lessons given by metabolic engineering, it follows that the key to creating biological control systems focused against HCC is precisely to learn about the molecular mechanisms that control the appearance and progression of this disease. Therefore, all strategies that seek to improve the treatments against HCC must consider the diverse internal (e.g., GRNs, mutations) and external factors (e.g., immune system activity) that can influence cell plasticity and could hinder the design work. However, a comprehensive understanding of all these factors represents a major challenge.

#### Physiological context of the liver

**FIGURE 2:**
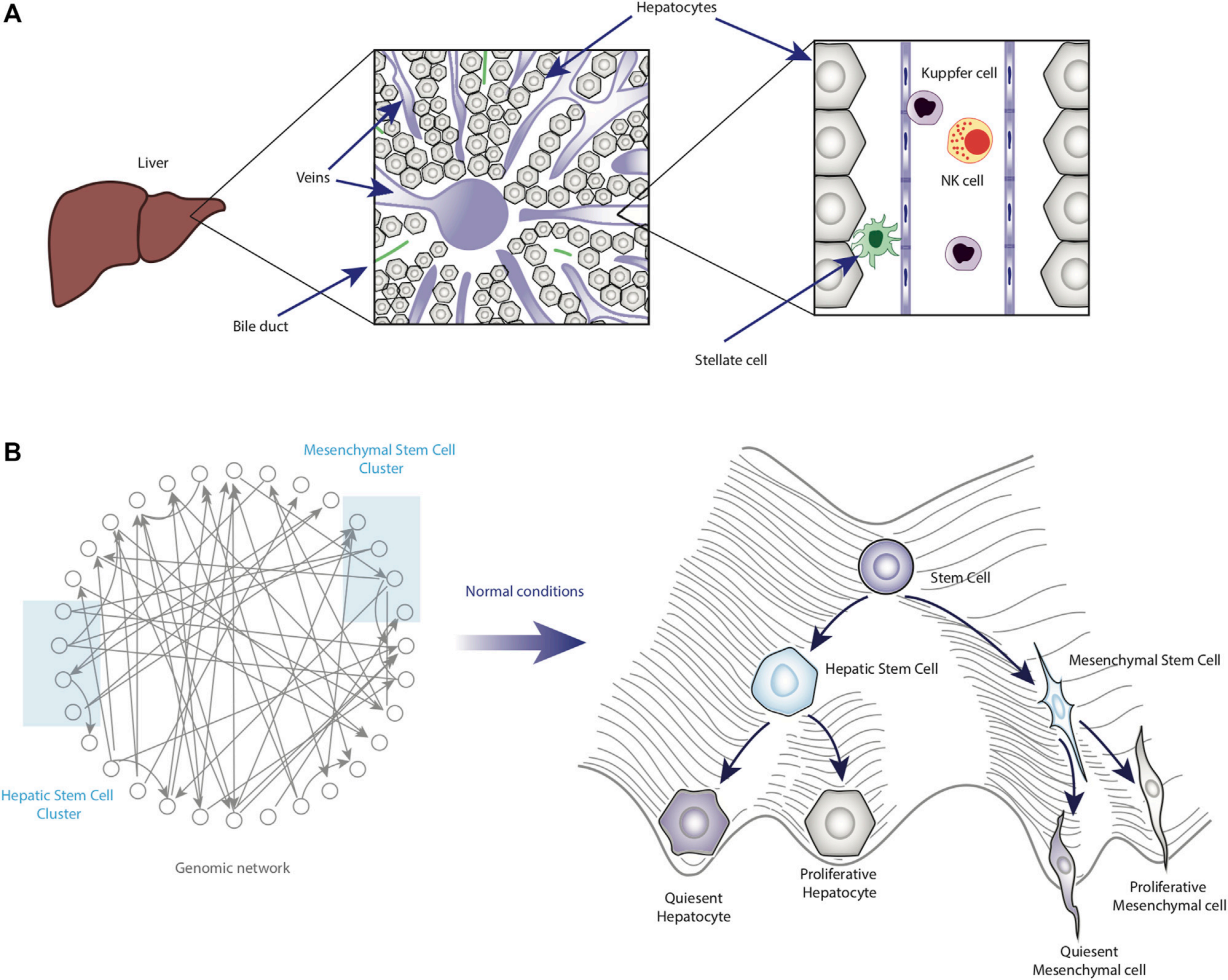
Homeostatic role of cell plasticity in the liver. Panel **(A)** shows a schematic representation of the liver under homeostatic conditions. Some of the main cell types are also shown in their anatomical location within the liver. Panel **(B)** shows the functioning and main features of the epigenetic landscape of normal liver cells.

Derived from the previous point, it is necessary to mention important aspects of liver physiology. In particular, this organ is composed of hepatocytes (epithelial cells that constitute more than 80% of the liver mass), cholangiocytes (biliary epithelial cells), endothelial cells, Kupffer cells, natural killer cells (NKs), and mesenchymal cells known as hepatic stellate cells (HSCs), whose chronic activation promotes liver fibrosis (Daoust and Cantero, 1959; Roskams, 2006; Higashi et al., 2017; Michalopoulos and Bhushan, 2021) (Figure 2A). Each cell type would correspond to a valley in the epigenetic landscape.

Considering the phenotype of hepatocytes in more detail, most hepatocytes are in quiescence (G0) and show a particular gene expression pattern including transcription factors such as HNF4a and HNF1a (Berasain and Avila, 2015). However, the proliferation of hepatocytes can be triggered by factors like liver injury, diminished cell density, or cell-cell interaction loss (Loyer et al., 1996). It has been reported that the co-culture of hepatocytes with cholangiocytes inhibits the response to growth factors (Loyer et al., 1996). Interestingly, hepatocytes in culture suffer dedifferentiation; it was observed that a stiff extracellular matrix promotes EMT and apoptosis resistance through the activation of Akt and ERK1/2 signaling (Godoy et al., 2009). However, these changes were reversible when hepatocytes were cultured in soft gel collagen (Godoy et al., 2009). Another study has shown that the transcriptional profile of primary cultured hepatocytes is similar to those of liver diseases such as cirrhosis and HCC (Godoy et al., 2016). Particularly, HNF4a downregulation has been related to the loss of specific cell-type features in cultured hepatocytes and HCC (Godoy et al., 2016; Lau et al., 2018).

Remarkably, liver cells exhibit outstanding phenotypic plasticity in response to injury. From the epigenetic landscape approach, these phenotypic transitions can be represented as jumps between valleys. Examples of this include the differentiation of liver progenitor cells (LPCs) into hepatocytes or cholangiocytes, as well as the differentiation of hepatocytes and cholangiocytes into LPCs, and the trans-differentiation between hepatocytes and cholangiocytes (Ko et al., 2020; Li et al., 2020; So et al., 2020). It has also been observed that HSCs show mesenchymal stem cell properties and can also give rise to hepatocytes and cholangiocytes (Kordes et al., 2015; Kordes et al., 2021). In addition, hepatocytes can undergo partial EMT during liver regeneration (Oh et al., 2018). Notably, these phenotypic transitions enable the robustness of liver functions against various types of damage. For example, in conditions of chronic liver damage, in which the population of hepatocytes has been severely impaired, the conversion of cholangiocytes into hepatocytes can contribute significantly to liver regeneration (Deng et al., 2018; Li et al., 2020).

Thus, understanding HCC requires considering the intrinsic plasticity that liver cells possess. In this sense, epigenetic landscape modeling could be essential to map the stable phenotypes emerging from the GRN (Figure 2B).

### Molecular bases of the EMT plasticity

Due to the relevance of EMT in the progression of HCC (Cicchini et al., 2015), we will focus our analysis on the regulation of this process. Molecularly, EMT plasticity is originated by several double negative feedback loops between epithelial and mesenchymal markers. For example, miR-200 and ZEB1, mir-34 as well as SNAIL, mir-200 and SNAIL (Garibaldi et al., 2012; Migault et al., 2022). Also, mutual negative regulation between SNAIL and HNF4A has also been identified in hepatocytes (Cicchini et al., 2015). Although these mutual inhibition interactions are related to the bistable behavior of EMT (Huang et al., 2017) they only partially control it. To understand exactly how EMT works, it is important to remember that this phenotypic transition is coupled to other cell processes like proliferation, senescence, stemness, and inflammation.

Multiple interactions have been reported among proliferation regulators such as β-catenin, YAP1, and c-Myc. Double negative feedback loops have been identified between Wnt- β-catenin signaling and HNF4A (Yang et al., 2013), as well as YAP1 and HNF4A (Cai et al., 2017). In addition, it has been found that the Wnt-β-catenin pathway positively regulates Snail (Jong et al., 2005) and mutual activation between YAP1 and SNAIL (Noce et al., 2019). It has also been reported that c-Myc positively regulates the SNAIL expression (Smith et al., 2009). HNF4A negatively regulates proliferation through repression of c-Myc and Cyclin D1 (Walesky and Apte, 2015). As mentioned above, the decreased expression of liver-specific genes such as HNF4A has been observed in cultured hepatocytes and HCC cells (Berasain and Avila, 2015). In this regard, over-expression of cell type-specific transcription factors decreased the efficiency of the dedifferentiation process induced by stem-related transcription factors in a neural-lineage cell line and hepatoblasts (Hikichi et al., 2013). In general, it is known that cell differentiation and the cell cycle are closely related through mutual inhibition between cell type-specific and cell cycle regulatory factors (Ruijtenberg and van den Heuvel, 2016; Zhao et al., 2020).

On the other hand, senescence has an influence on EMT and vice versa (Kishi et al., 2015). p53 activates the expression of miR200c and mir34a, which are associated with the conservation of the epithelial phenotype (Bommer et al., 2007; Chang et al., 2011). The loss of p53 decreases the expression of miR200c and mir34a and it induces EMT (Chang et al., 2011; Kim et al., 2011). At the post-transcriptional level, SNAIL and p53 negatively regulate each other (Lee et al., 2009; Lim et al., 2010; Ni et al., 2016). Moreover, it has been reported that SLUG suppresses the expression of p16 (Zhu et al., 2019).

The relationship between EMT and stemness has been also studied. Although OCT4 and NANOG activate SNAIL expression to induce EMT (Yin et al., 2015), the regulation is not simple since NANOG can also activate SLUG expression (Yao et al., 2016) and SNAIL can suppress NANOG expression promoting stem cell differentiation (Galvagni et al., 2015). In hepatocytes, HNF4 and SNAIL control the expression of stem markers through the regulation of miR-200 and miR-34a (Garibaldi et al., 2012). It is interesting that it has been reported that OCT/SOX2 activate miR200s family expression during fibroblast reprogramming (Wang et al., 2013) and that the miR-200 family negatively regulates SOX2 and KLF4 (Wellner et al., 2009; Pandey et al., 2015). It is known EMT plays a role in the emergence of cancer stem cells (Mani et al., 2008; Morel et al., 2008; May et al., 2011; Andriani et al., 2016; Jayachandran et al., 2016; Harner-Foreman et al., 2017; Jing et al., 2019) and in the reprogramming of somatic cells (Esteban et al., 2012; Shu and Pei, 2014).

Finally, experimental evidence shows that inflammation and EMT are also closely coordinated (López-Nouoa and Nieto, 2009). In particular, the IL-6/STAT3 pathway and HNF4A negatively regulate each other through miRNAs (Yan et al., 2018). Notably, a miRNA-mediated mutual inhibition circuit was also reported between NF-κB and HNF4A (Ning et al., 2014). Conversely, NF-κB activates the expression of mesenchymal markers such as SNAIL, SLUG, ZEB1, and TWIST (Min et al., 2008). Since HCC and other types of cancer arise and develop in a chronic inflammation context, the influence of inflammation on EMT and other processes is especially important (Taniguchi and Karin, 2018; Yan et al., 2018; Czauderna et al., 2019).

Collectively, these interactions highlight the tremendous complexity of the intracellular regulatory network underlying the EMT plasticity. In this context, mathematical-computational modeling has provided a framework to integrate the vast amount of available information and explain some aspects of cell plasticity, as we will see in the next sections.

### Cancer stem cells are originated by plasticity

**FIGURE 3:**
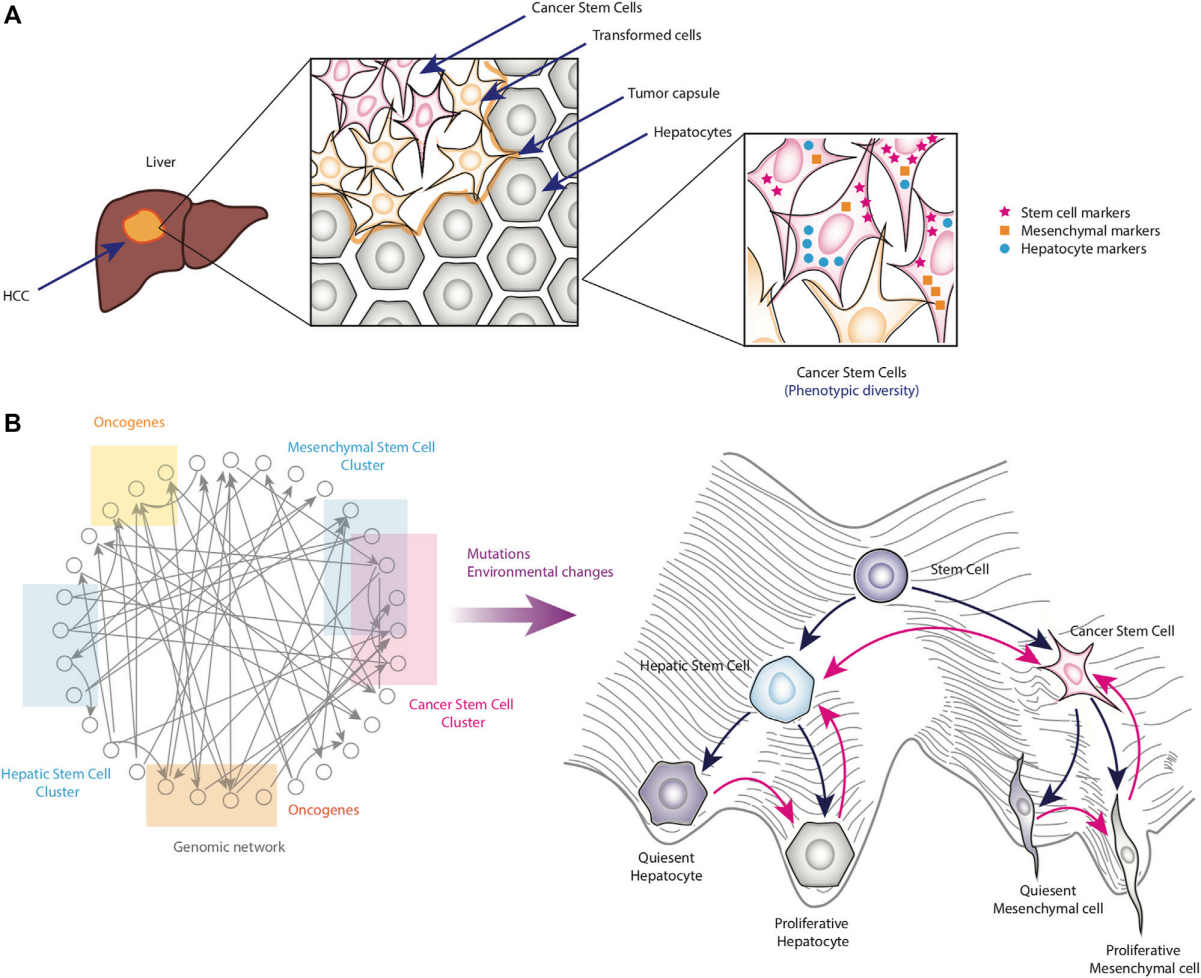
Emergence of CSCs due to cell plasticity alterations. Panel **(A)** shows a schematic representation of the anatomy of a tumor produced by HCC. This tumor shows the niche of the CSCs where they are protected from the external environment. Of importance is that the CSCs are heterogenous at phenotypic level, since they may present different expression levels of stem-cell markers (pink stars), hepatocyte markers (blue dots) or mesenchymal markers (orange squares). Panel **(B)** shows a representation of the effect of mutations and disturbances produced by the microenvironment on the topology of the epigenetic landscape. Collectively, those perturbations transform the topology of the epigenetic landscape to generate altered phenotypes such as CSCs (pink arrows).

At this point it is clear that there is enough information to construct GRNs models. However, it is important to define and identify the types of tumor cells to be studied (Figure 3A). In this direction, tumors can be phenotypically heterogeneous, which means that tumor cells may exhibit variations in phenotypic traits such as gene expression, metabolism, motility, stemness, proliferation, and metastatic capacity (Marusyk and Polyak, 2010).

In particular, tumor cells that present stem-like phenotypes, known as cancer stem cells (CSCs), are being intensively investigated. Notably, such cells present subpopulations that show different expression levels of stemness, epithelial and mesenchymal markers, and different proliferative capacities (Mohan et al., 2021). In this regard, phenotypic heterogeneity has important functional implications: quiescent CSCs or CSCs with a low cell division rate are related to therapy resistance and recurrence due to most chemotherapeutic agents target the proliferative cells, while CSCs with a hybrid epithelial and mesenchymal phenotype are highly prone to initiate metastasis (Mohan et al., 2021). Interestingly, the frequency of CSCs has been observed to increase according to the stage of tumor development (Yamashita and Wang, 2013).

Although several sources of intratumoral heterogeneity have been identified, including mutations, molecular epigenetic changes, and signals from the microenvironment (Marusyk et al., 2020), these factors are frequently studied separately, which may limit the understanding of intratumoral heterogeneity and its implications. In this sense, the epigenetic landscape approach could serve as an integrative framework.

Typically, somatic mutations are assumed to be the major source of intratumoral heterogeneity while network dynamic that controls phenotypic plasticity is ignored. In this regard, the integration of genetic and non-genetic mechanisms that control cell plasticity could give us a greater understanding of tumor cell plasticity and intratumoral heterogeneity (Pisco and Huang, 2015; Huang, 2020). It has been suggested that mutations may alter the topography of the epigenetic landscape, potentially changing the barriers between different valleys. This, in turn, could modify the probabilities of phenotypic transitions (Figure 3B) (Huang, 2013; Huang, 2020). Considering the transition into stem-like states, experimental observations might agree with this. For instance, it has also been observed that normal, immortalized, or transformed human breast epithelial cells can convert into CD44^+^ cells, which is a marker of stemness (Chaffer et al., 2011). However, transformed cells overexpressing RAS, telomerase, and a viral oncoprotein show an increased ability to become stem-like cells compared to non-transformed cells (Chaffer et al., 2011).

At least two mechanisms have been proposed for CSCs emergence in HCC: the transformation of liver progenitor cells and the differentiation of epithelial cells (Nio et al., 2017; Yagci et al., 2017; Wu et al., 2020). Indeed, several results indicate that the appearance of stem-cell markers is a result of EMT in HCC and other cancer types (Giannelli et al., 2016; Chen et al., 2017; Forte et al., 2017; Jing et al., 2019). For instance, Mani *et al* demonstrated that EMT can be induced either by TWIST, SNAIL ectopic expression or by TGF-β treatment, in immortalized human mammary cells, promoted the increase of the subpopulation CD44^+^. Remarkably, such cells were able to form mammospheres whereas CD44^−^ cells were unable to do so (Mani et al., 2008).

On the other hand, environmental factors such as inflammation and hypoxia also promote the acquisition of stem cell traits (Figure 3B). IL-6 treatment induces EMT and the acquisition of stem cell properties in breast cancer cells (Xie et al., 2012). Similarly, IL-6 secreted by tumor-associated macrophages promoted the expansion of the CD44^+^ cell population in HCC (Wan et al., 2014). Also, hypoxia upregulates OCT4, NANOG, and BMI-1 in HCC and HIF-1α knockdown inhibited hypoxia-induced stem-like properties (Cui et al., 2017). It is of interest that both IL-6 and IL-1β upregulate HIF-1α expression (Wan et al., 2014; Zhang et al., 2018). Complementary observations obtained from modelling of EMT and stemness modules indicate that NF-κB might stabilize the epithelial-mesenchymal hybrid phenotype and increase the likelihood of gaining stem-like properties (Jolly et al., 2014).

On the whole, these data suggest that there are three main stable states: epithelial cells, mesenchymal cells, and stem-like cells, that must be considered to explore the phenotypic plasticity involved in CSC emergence. Using a quantitative epigenetic landscape model, one would identify how mutations and environmental signals specifically change phenotypic transition probabilities. Similarly, targets could be found that inhibit undesirable phenotypic transitions.

#### Cancer-associated senescence is a plastic state

In addition to the interactions and the characterization of the stable states of a network, it is necessary to know if there is evidence that proves that the system can leave or transit toward a state of interest. We will examine the case of senescence for its relevance in cancer progression.

Traditionally, senescence has been defined as a state of the permanent arrest of the cell cycle induced by several types of stress, such as severe DNA damage or oncogene activation (Herranz et al., 2018; Takasugi et al., 2022). Furthermore, senescent cells are unresponsive to growth factors, resistant to apoptosis, and secrete a mix of proinflammatory cytokines, chemokines, growth factors, and proteases called senescence-associated secretory phenotype (SASP) (Herranz et al., 2018; Paez Ribes et al., 2019). Senescence is induced in response to various stressors and prevents the proliferation of damaged cells, thus, it is considered a tumor suppressor mechanism. However, the accumulation of senescent cells in tumors may also favor a pro-inflammatory and pro-proliferative microenvironment *via* the SASP (Herranz et al., 2018). It suggests the manipulation of senescence must rely on context.

Although the senescent state is a very stable phenotype, the transition from senescent phenotype to proliferative phenotype has been observed experimentally (Lee and Schmitt, 2019; Saleh et al., 2019; Walcher et al., 2020). In particular, it has been reported that colon cancer cells with p53 and p16 inactivation were able to enter senescence (Carné Trécesson et al., 2011). Nevertheless, these senescent cells resumed proliferation after 4–5 weeks. Interestingly, such cells showed upregulation of vimentin and downregulation of E-cadherin, which is associated with EMT induction (Carné Trécesson et al., 2011).

In a similar way, senescent fibroblast attained escape from oncogene-induced senescence after a prolonged period in culture, because of telomerase reactivation (Patel et al., 2016). It is interesting that senescence escape of lymphoma cells has been associated with acquiring stem cell features (Milanovic et al., 2018). Remarkably, the reprogramming of senescent fibroblasts into induced pluripotent stem cells using OCT4, SOX2, KLF4, c-Myc, NANOG, and LIN28 has been experimentally demonstrated (Lapasset et al., 2011).

**FIGURE 4:**
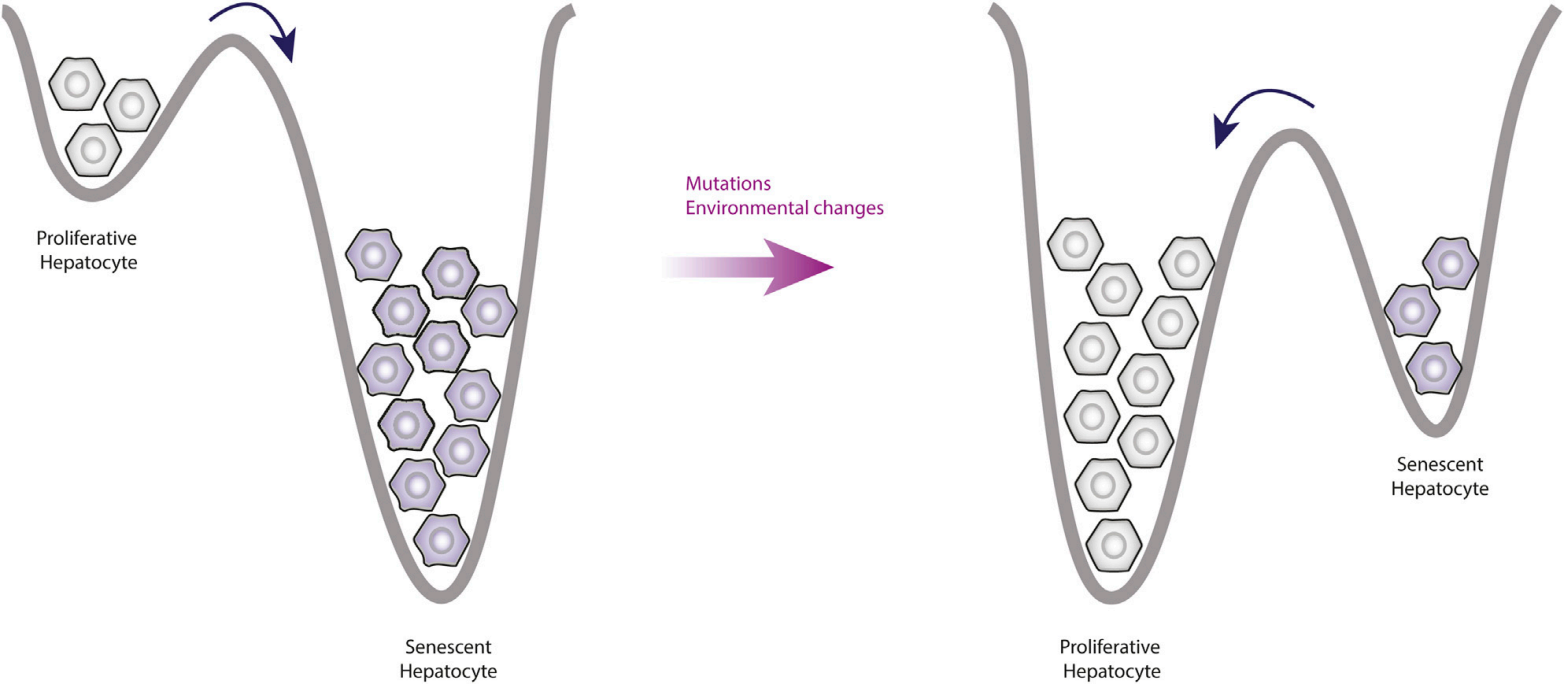
Observable changes produced by alterations of the epigenetic landscape. This figure shows a simplification of the epigenetic landscape in two dimensions. Under normal conditions, the valleys of senescent phenotypes are deeper than the valleys of proliferative phenotypes, this means that the proliferative cells tend to enter senescence more easily. However, mutations affect the topology of the landscape by increasing the valleys depth of proliferative phenotypes, which means that there will be more cells going into proliferation and leaving senescence.

Together, these data indicate that even states like senescence can be reversible through manipulation (Figure 4). Importantly, the immune system is responsible for removing the senescent cells (Herranz et al., 2018), but it also plays a relevant role on EMT.

### Effect of perturbations produced by the immune system on EMT

**FIGURE 5:**
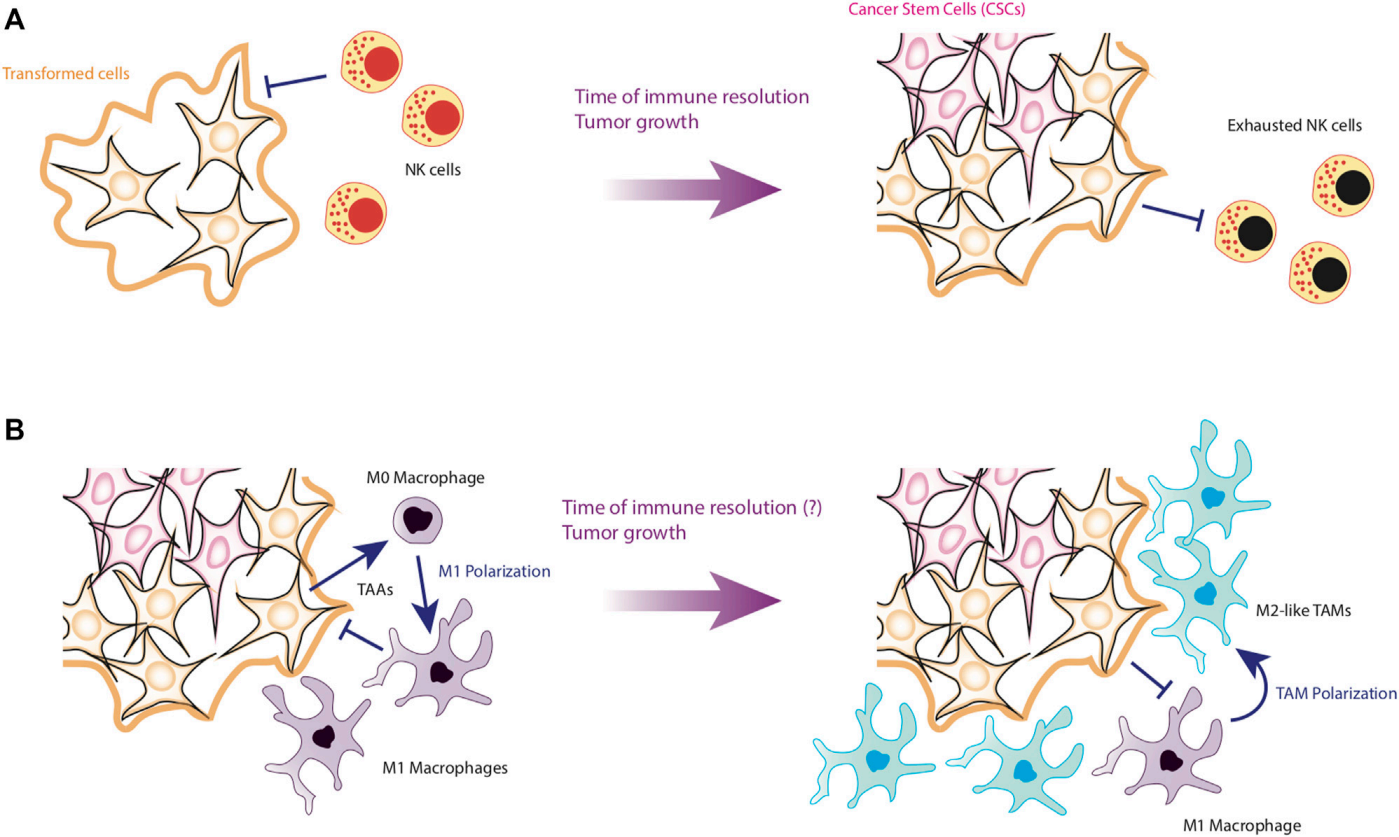
First stages of HCC development. Panel **(A)** shows the early stages of HCC formation. NK cells are able to efficiently detect and eliminate transformed cells by apoptosis or by the release of Granzyme **(B)**. However, if NK are slow to kill tumor cells, NK cells will be exhausted and allow the emergence of CSCs. Panel **(B)** shows the establishment of the tumor microenvironment. After the formation of CSCs, the internalization of macrophages is favored, which can still kill tumor cells. However, if they fail to kill the tumor in a short time, it is possible that the differentiation of M1 macrophages towards the TAM phenotype is induced, favoring the establishment of a pro-tumorigenic microenvironment.

Finally, it is necessary to put into perspective the effect of perturbations produced by external factors involved in the generation of cancer. In this direction, the immune system is essential to determine cancer progression. To be precise, recent evidence suggests that the main factor against tumor recovery is the time it takes for the immune system to control the neoplasia. For instance, it has been observed that the longer it takes NK cells to kill cancer cells, the more likely they are to survive (Ichise et al., 2022) (Figure 5A). Additionally, if the immune system takes longer to eliminate tumor cells, CCL2 levels increase in the tumor microenvironment (Qiu et al., 2018), which will favor macrophage infiltration. This is particularly important for HCC, since the immune cells with the greatest presence in the liver are precisely NK cells (Sajid et al., 2022).

In this scenario, the chances of newly infiltrating macrophages becoming tumor-associated macrophages (TAMs) are increased due to interactions with cancer cells and tumor microenvironment (Zhou et al., 2020) (Figure 5B). Concerning TAMs, they present two main variants, the M1-TAM phenotype and the M2-TAM phenotype (Arvanitakis et al., 2022). Like their non-tumor-associated counterparts, M1-TAMs promote tumor elimination while M2-TAMs produces an anti-inflammatory microenvironment, which promotes tumor progression and metastasis (Arvanitakis et al., 2022). TAMs make up more than 50% of the cells that make up the periphery of tumors (Azizi et al., 2018), and depending on the prevalence of the subtype of TAMs, the tumor may compromise the life of the patient. Specifically, a high prevalence of M2-TAMs is associated to bad prognosis for patients (Arvanitakis et al., 2022).

**FIGURE 6:**
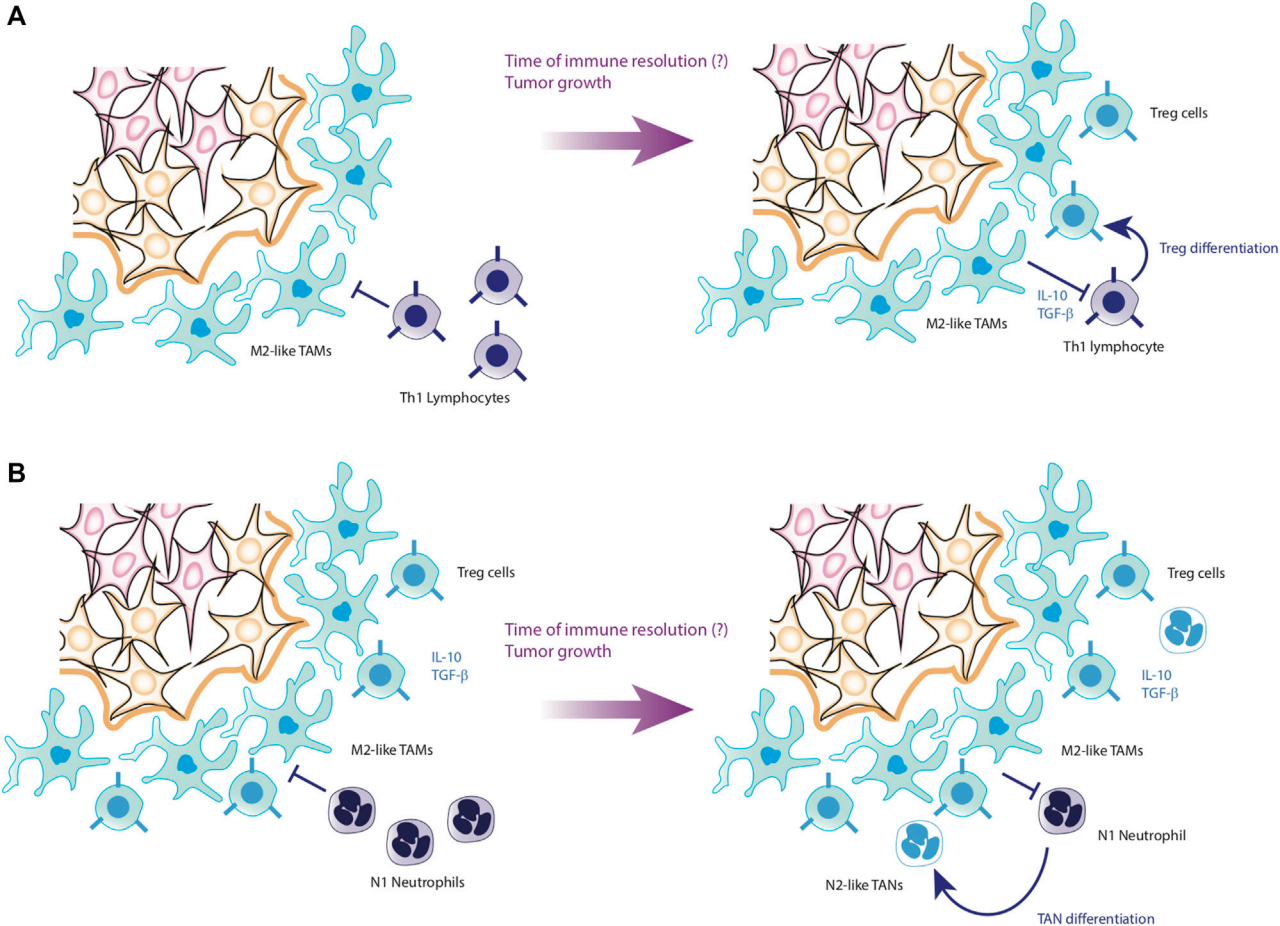
Progression of HCC to metastasis. Panel **(A)** shows the conversion process of Th1 cells into Treg cells. Through the anti-inflammatory cytokines secreted by TAMs, Th1 cells lose their effectiveness in fighting the tumor, joining the tumor microenvironment as Treg cells. Panel **(B)** shows the last step before starting metastasis. In case that T lymphocytes cannot neutralize the tumor, the last cells to participate are the neutrophils, which, due to the action of the microenvironment formed by tumor cells, TAMs and Treg cells, end up joining the tumor microenvironment in the form of TANs. Hereafter, it is not known exactly what causes TANs to secrete their NETs, favoring the detachment of tumor cells to initiate metastasis.

The importance of TAMs in the maintenance of tumors is mainly due to the fact that they produce an anti-inflammatory microenvironment rich in cytokines such as IL-10 and TGF-β, while these macrophages maintain low levels of proinflammatory cytokines such as TNF-α, IL-12, IL-6 and IL-1β (Feng et al., 2022). As a consequence, it has been reported that tumor microenvironment severely depletes the effector phenotypes of the immune system, causing both CD4^+^ T cells and CD8^+^ T cells to become anergic and express surface markers such as PD-1, facilitating their clearance (Ma et al., 2019; Di Blasi et al., 2020). On the other hand, TAMs also activate the growth pathways of tumor cells, maintaining an optimal microenvironment for their proliferation. Therefore, TAMs also induce EMT of epithelial cells, promoting their malignant transformation (Gifford et al., 2017; Ran et al., 2021). Furthermore, TAMs directly kill effector cells express the death ligands FasL and PD-L1 on their membranes, which induce apoptosis in effector cells (Feng et al., 2022) (Figure 6A).

Moreover, the sustained activity of TAMs converts effector immunological cells into regulatory phenotypes unable to inhibit the tumor. TAMs also promote the internalization of Myeloid inhibitory cells (MDSCs), which enhances the anti-inflammatory microenvironment and proliferation of the tumor through the activation of growth pathways (Pan et al., 2020). As a consequence of this prolonged stimulation, tumor cells increase the secretion of CXCL5 (Zhou et al., 2016), which is a localization signal for neutrophils. Once the neutrophils infiltrate in the affected tissue and interact with TAMs as well as the tumor microenvironment, they loss their effector phenotype and become tumor-associated neutrophils (TANs) (Wu and Zhang, 2020).

Analogous to TAMs, TANs remain at the periphery of the tumor, although the increase in its presence is negatively correlated with patient survival. The TANs, like the TAMs, also have N1-TAN and N2-TAN phenotypes, the latter being anti-inflammatory in nature and particularly harmful to the patient’s life (Arvanitakis et al., 2021). Recent research found a pernicious dynamic caused by TANs. First, it has been documented that N2-TANs positively regulate the appearance of regulatory phenotypes of CD4^+^ T lymphocytes by secreting CCL17 (Shaul et al., 2016). Likewise, it has been observed that TANs promote the appearance of TAMs and promote the growth of cancer cells, which generates an increase in CXCL5, proliferating the presence of TANs in the tumor with a positive feedback loop (Zhou et al., 2016). The increase in TANs produces the release of Neutrophil Extracellular Traps (NETs), which promote the detachment of tumor cells from the tissue (Masucci et al., 2020), and together with the increase in the expression of VEGF and other angiogenic signals by TAMs (Pan et al., 2020), gives rise to tumor metastasis (Figure 6B). In particular, it has been seen that the infiltration of TANs is very strong in the context of HCC, which associates these cells directly with the progression and worsening of the clinical condition of the patients (Zhou et al., 2016).

### State of the art of cancer modeling

The data collected herein demonstrate that there is sufficient information to create models that adequately represent EMT. However, it remains to be determined whether there are tools capable of capturing all aspects needed to design therapeutic strategies against HCC. In this regard, a computational model provided by Chong *et al* proposed a GRN model in which they identified three attractors: homeostasis, cell cycle arrest, and senescence. It is of interest that the senescence phenotype was favored, as occurs experimentally (Chong et al., 2018). This model showed that p53 inactivation, in presence of DNA damage, modifies the epigenetic landscape structure and allows the emergence of a new attractor related to a cancer state (Chong et al., 2018). In the same line, Méndez-López and coworkers proposed a model of EMT in which they identified attractors that correspond to epithelial, senescent epithelial, and stem-like mesenchymal phenotypes. In this model, the loss of function of p16 abrogates the senescent epithelium attractor. Furthermore, the results suggested that senescent epithelial cells might exit the senescent state and acquire mesenchymal stem cell characteristics, as has been observed in spontaneous immortalization (Méndez-López et al., 2017).

Concerning to the immune system, computational models have been used to improve detection of tumors using IgG and IgM antibodies (Díaz-Zaragoza et al., 2015). Also, models of multi-scale agents have been created to simulate the interactions between CD4^+^ T cells and macrophages in the tumor microenvironment (Cess and Finley, 2020). In the same direction, models based on Boolean GRNs have been made to understand the fine details of macrophage differentiation and formation of TAMs in the context of the tumor microenvironment (Avila-Ponce de León et al., 2021). These models have shown that the phenotypic diversity of TAMs can be explained by a sensitive balance produced by transcriptional factors such as NF-κB, STAT3, STAT6, AP-1 and HIF-1α (Avila-Ponce de León et al., 2021). However, none of the aforementioned models has integrated by itself the complexity of cancer, in particular specialized aspects such as those seen in HCC.

## Discussion

Currently, there is no single way to design therapeutic strategies against cancer, since all approaches, to the extent of their possibilities, may contribute against this disease. However, it is tempting to assume that it is possible to create rational therapeutic strategies. Especially if we consider the difference in mortality associated with different types of cancer, such as HCC (Rumgay et al., 2022). Furthermore, it would be extremely useful to be able to design personalized therapeutic strategies at low cost for each patient. In this direction, engineering shows that it is possible to create therapeutic strategies as long as two conditions are met: first, the behavior of the system to control can be visualized and second, the system must be controllable (Mellodge, 2015). Regarding the first condition, in the present work we have found ample experimental evidence that demonstrates that there is enough data to create models capable of visualizing the complexity of cancer, including specific types of cancer such as HCC. Concerning the second condition, the experimental evidence cited in this work proves that it is possible to guide cancer cells towards diverse phenotypes. In other words, it is theoretically possible to create therapeutic strategies using rational engineering principles to control cancer.

Consequently, a reasonable question is why this approach is not currently applied to the clinic. Although hypothetically this approach can be used, the truth is that there are several technical limitations that make its clinical implementation difficult. Firstly, even though there is sufficient information to model the complexity of cancer, the use of GRNs models requires a large number of biological components, which implies a high computational cost. Secondly, it is necessary to know all the possible side effects of the drugs used and genetic circuits in the design of therapeutic strategies. Regarding the first limitation, characterizing multistability requires excessive computational effort. To illustrate this point, suppose that a network of 30 nodes is being analyzed, this means that the number of states that the network can originate is $2^{30}=1,073,741,824$ . But, if instead of 30 nodes, 40 nodes were required to make a better model, then the size of the state space increases to $2^{40}=1,099,511,627,776$ . Considering this example, it is easy to understand what the result would be of making detailed models that include components of epithelial cells as well as immune system networks.

Nevertheless, there are advances in computer science that could solve the state space size problem. In particular, the methodology known as Process Hitting appears to be a viable solution to the problem, since it is capable of obtaining information from immensely large networks without being limited by the problem of the size of the state space (Folschette et al., 2015; Sheikh et al., 2019). Although this technique is still being experimented, it is not ruled out as a viable solution in the medium-term (Sheikh et al., 2019). Regarding the side effects of drugs, at the moment it is impossible to predict their behavior before clinically testing. However, databases of side effects such as SIDER (Kuhn et al., 2016) could help to select drugs to be considered in the design of therapeutic strategies.

In conclusion, from everything discussed up to this point, it is clear that the rational design of therapeutic strategies against liver cancer, as well as for other types of cancer, could be achieved with sustained efforts in the medium-term. As a fundamental condition for this, it is necessary to deal with the problem of the state space of the GRNs and in addition, the built models must necessarily consider the interactions of cancer cells with their microenvironment. This implies including models of the immune cells that constitute the periphery of the tumors, as well as the cancer cells in their different phenotypes inside the tumors.
